# Association between red and processed meat intake and colorectal adenoma incidence and recurrence: a systematic review and meta-analysis

**DOI:** 10.18632/oncotarget.23561

**Published:** 2017-12-21

**Authors:** Zhanwei Zhao, Zifang Yin, Zhenning Hang, Chaojun Zhang, Qingchuan Zhao

**Affiliations:** ^1^ Department of Surgery, Navy General Hospital of PLA, Beijing, China; ^2^ Xijing Hospital of Digestive Diseases, The Fourth Military Medical University, Xi'an, China; ^3^ Department of Obstetrics, Northwestern Women and Children's Hospital, Shaanxi Province, China

**Keywords:** meta-analysis, red meat, processed meat, colorectal adenoma, recurrence

## Abstract

The associations between red and processed meat intake and colorectal adenoma (CRA) incidence and recurrence are inconclusive. We performed a systematic review and meta-analysis to analysis these associations. We conducted a systematic search of PubMed, EMBASE and Web of Science up to December 2016. The relative risks (RRs) and 95% confidence intervals (CIs) were assessed. Subgroup analyses, dose-response-analyses, subtype analyses and analyses of CRA locations were also conducted. Twenty-seven studies that involved 208,117 participants and 19,150 cases met criteria. The RRs of the highest versus lowest intakes for CRA incidence were 1.23 (1.15–1.31) for red meat and 1.15 (1.07–1.24) for processed meat. Dose-response analyses for meat per 100 g/day yielded the results were consistent with the original analyses, with 1.14 (1.07–1.20) for red meat and 1.27 (1.03–1.50) for processed meat. Additionally, there were no associations between red and processed meat intake and CRA recurrence, including total CRA (*P* > 0.05), advanced CRA (*P* > 0.05) and multiple CRA (*P* > 0.05). In conclusion, our findings support the hypothesis that red and processed meat intake was associated with an increased CRA incidence but not for CRA recurrence.

## INTRODUCTION

According to the Cancer Statistics 2017, colorectal cancer (CRC) is the third most frequently diagnosed cancer, with 135,430 estimated new cases and 50,260 estimated deaths in 2017 occur in the United States [1]. The adenoma-carcinoma sequence represents the process by which most CRC has increased [2]. Thus, focusing on CRA risk factors is important to enhance our understanding of colorectal carcinogenesis. Recently, an increasing number of studies have focused on dietary factors [3, 4]. The continuously updated project report of the World Cancer Research Fund/American Institute for Cancer Research (WCRF/AICR) has classified red and processed meat intakes as “convincing evidence” for CRC [5, 6]. However, the associations between red meat and processed meat intake and CRA risk have been unclear. Two systematic analyses [7, 8] on the associations have been reported worldwide in which studies published up to 2011 were included, and showed that increased intake of red and processed meat was associated with increased CRA risk. Nevertheless, several high-quality studies [9–11] have appeared during the last 5 years (approximately) and did not support the conclusion of the systematic analyses. An updated meta-analysis of the literature could clarify the impact of these recent studies. Furthermore, no systematic review or meta-analysis has been performed to assess the association between red and processed meat intake and colorectal adenoma recurrence to date. Thus, considering the high incidence and fatality of CRC and the limited evidence of CRA, we performed a systematic review and meta-analysis with the following objectives: (1) to evaluate the associations between red and processed meat intake and CRA incidence and recurrence; (2) to assess the dose-response associations between red and processed meat intake and CRA risk; and (3) to further provide detailed subgroup analyses of studies and evidence according to subtype analyses of meat.

## RESULTS

### Literature selection, study characteristics and quality scores

**Figure 1:**
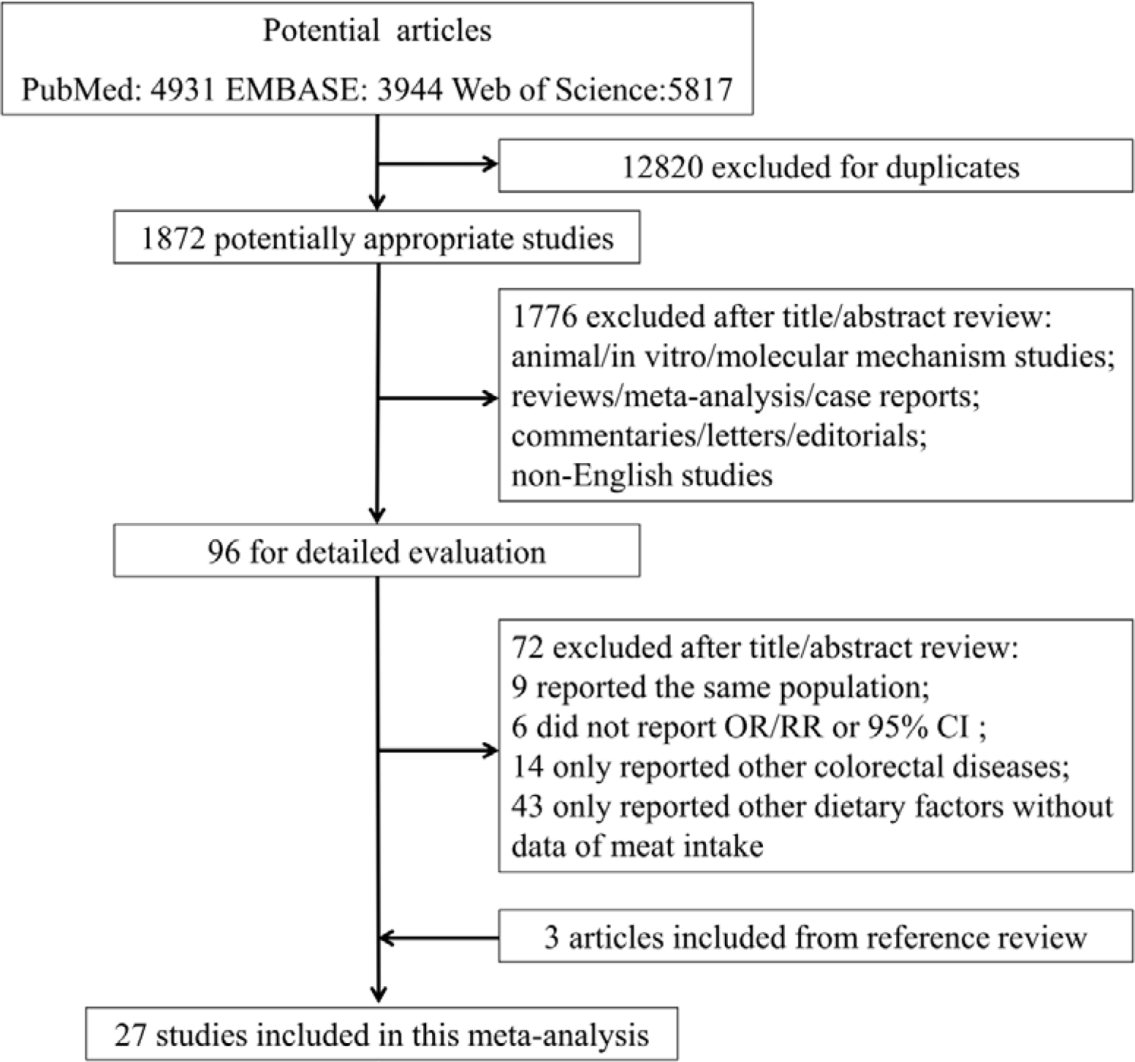
Flowchart of process for identification of relevant studies

Twenty-seven studies met the criteria and provided 34 separate estimates (red meat, 24; and processed meat, 10) for CRA incidence, and 20 separate estimates (red meat, 10; and processed meat, 10) for CRA recurrence (Figure 1). The included studies were from 9 countries or regions in America, Europe and Asia with 208,117 participants and 19,150 cases. The NOS scores ranged from 6 to 9 (Table 1) [9–35].

**Table 1:** Baseline characteristics of included studies for meat intake and colorectal adenomas risk

First author, year, country	Study design	Case/control (cohort, n)	Study period	Type of dietary exposure	Dietary exposure categories	Adjusted RRs (95% CI) (highest to lowest)	Adjusted variables	NOS score
Giovannucci 1992 USA [12]	co	170/7284	1986–1988	Red meat	Quintile	1.23 (0.70–2.14)	Age, total energy intake, family history of CRC	7
Sandler 1993 USA [13]	cc	236/409	1988–1990	Beef	Quintile	1.78 (0.97–3.27)	Age, alcohol, BMI, calories	6
Haile 1997 USA [14]	cc	488/488	1991–1993	Beef Processed meat	Quintile	1.83 (1.12–2.99) 1.48 (0.92–2.39)	Age, gender, NSAIDs use, fat, vegetable, protein, carbohydrates, fiber, cholesterol, BMI, physical activity, calories, smoking, ethnicity	7
Lubin 1997 Israel [15]	cc	196/196	1979–1989	Beef	Tertile	1.60 (0.90–2.70)	Energy intake and physical activity	6
Breuer-Katschinski 2001 Germany [16]	cc	184/184	1993–1995	Beef	Quintile	3.10 (1.46–6.43)	Energy, relative weight and social class	6
Nagata 2001 Japan [17]	co	279/28361	1992–1995	Beef and pork	Middle vs highest	1.06 (0.77–1.46)	Age, total energy, smoking, alcohol	6
Voskuil 2002 Netherlands [18]	cc	119/148	1995–1998	Red meat	Tertile	1.20 (0.12–12.00)	Age, gender, energy intake	6
Tiemersma 2004 Netherlands [19]	cc	431/433	1997–2000	Red meat	Quartile	1.20 (0.80–1.80)	Age, gender, and indication of endoscopy	7
Chan 2005 USA [20]	ncc	527/527	1976–1990 1990–1998	Red meat	Quartile	1.57 (0.93–2.65)	Age, fasting status, date of blood draw, history of previous endoscopy, BMI, smoking, physical activity, calcium, folate, alcohol, multivitamins, aspirin, menopause status	8
Sinha 2005 USA [21]	ncc	3498/34817	1993–2001	Red meat Processed meat	Quintile	1.07 (0.92–1.24) 1.04 (0.90–1.19)	Age, gender, screening center, energy intake, ethnicity, education, tobacco use, alcohol, use of aspirin and ibuprofen separately, physical activity, total folate intake, calcium intake, dietary fiber intake	9
Wu 2006 USA [22]	co	581/14032	1996–2002	Red meat Processed meat	Quintile	1.18 (0.87–1.62) 1.52 (1.12–2.08)	age, family history of CRC, reason for endoscopy, negative endoscopy before 1996, physical activity, smoking, race, aspirin use, total energy intake, calcium and folate intake	8
Cho 2007 USA [23]	co	2408/39246	1984–2002	Red meat	Quintile	1.41 (1.11–1.79)	age, smoking, BMI, physical activity, family history of CRC, history of endoscopic screening, year of endoscopy, aspirin use, menopausal status and HRT, energy intake, alcohol, folate, total fiber and calcium	9
Saebo 2008 Norway [24]	cc	422/222	1995–1999	Red meat	Tertile	1.22 (0.78–1.91)	Age, gender	6
Ferrucci 2009 USA [25]	cs	158/649	2000–2002	Red meat Processed meat	Quartile	2.02 (1.06–3.83) 1.05 (0.59–1.85)	Age, education, race, smoking, physical activity, BMI, study center, HRT, family history of colorectal polyps or CRC, NSAIDs use, alcohol, fiber, calcium, total caloric intake	7
Ramadas 2009 Malaysia [26]	cc	59/59	Jan-Dec 2005	Red meat	≥ 3 vs. < 3 times/week	2.51 (1.00–6.28)	Age, ethnicity, gender, physical activity, height, BMI, waist circumference, energy intake, drinking and smoking	6
Rohrmann 2009 Europe [27]	co	516/25540	1998–2007	Red meat	Quartile	1.33 (0.95–1.85)	Energy intake without energy from alcohol, ethanol intake, milk and milk product, fiber, BMI, family history of CRC, physical activity, NSAID, smoking, education, age and sex	8
Northwood 2010 UK [28]	cc	317/296	No	Red meat	Quartile	0.85 (0.53–1.36)	Age and sex	6
Wang 2011 USA [29]	cc	914/1185	1995–2007	Red meat Processed meat	Tertile	1.11 (0.83–1.48) 1.23 (0.94–1.61)	Age, sex, ethnicity, daily energy intake, physical activity, recruitment site and examination procedure, BMI, smoking, alcohol, folate	8
Burnett-Hartman 2011 USA [30]	cc	519/772	2004–2007	Red meat	Tertile	1.19 (0.80–1.78)	Age, gender, race, education, BMI, alcohol, NSAIDs use, HRT	8
Fu 2011 USA [31]	cc	1881/3764	2003–2010	Red meat Processed meat	Quartile	1.40 (1.20–1.60) 1.30 (1.10–1.50)	Age, sex, race, study site, education, indications for colonoscopy, smoking, alcohol, BMI, physical activity, regular NSAIDs use, total energy intake, recruitment before or after colonoscopy	9
Ferrucci 2012 USA [32]	co	1008/17072	2001–2009	Red meat Processed meat	Quartile	1.22 (0.98–1.52) 1.23 (0.99–1.54)	age, study center, gender, ethnicity, education, family history of CRC, BMI, NSAID use, physical activity, smoking, alcohol, supplemental calcium, dietary fiber, total energy intake	9
Nimptsch 2013 USA [11]	co	1494/19771	1998–2007	Red meat Processed meat	Quartile	0.96 (0.74–1.23) 0.92 (0.76–1.11)	age, family history of CRC, endoscopy, height, BMI, smoking, physical activity, aspirin use, high school/adult energy intake, alcohol	9
Cross 2014 USA [10]	cc	131/131	1994–1996	Red meat Processed meat	Quartile	1.40 (0.66–2.96) 0.98 (0.43–2.23)	Age, sex, education, race, BMI, family history of CRC, smoking, physical activity, fiber intake	7
Budhathoki 2015 Japan [9]	cc	738/697	2004–2005	Red meat Processed meat	Quartile	1.19 (0.87–1.63) 1.28 (0.92–1.78)	Age, screening period, smoking, alcohol, BMI, physical activity, family history of CRC, NSAIDs use. Further adjusted for age at menopausal status, and HRT in women	8
Mathew 2004 USA [33]	RCT recurrence	958/947	1994–1998	Red meat Processed meat	Quintile	0.98 (0.71–1.35) 0.92 (0.68–1.25)	age, sex and group	
Robertson 2005 USA [34]	co recurrence	539/1519	1984–1988	Red meat Processed meat	Quartile	0.97 (0.78–1.21) 1.15 (0.92–1.43)	age, sex, clinical center, treatment category, study, the duration of the observation period	8
Martinez 2007 USA [35]	RCT recurrence	379/869	1995–1999	Red meat Processed meat	Tertile	1.06 (0.72–1.55) 1.29 (0.89–1.86)	age, sex, previous polyps and number of colonoscopies during follow-up	

CRC: colorectal cancer; RCT: randomized controlled trial; co: cohort; ncc: nested case-control; cc: case-control; cs: cross-sectional; RRs: relative risks; 95% CI: 95% confidence intervals; BMI: body mass index; NSAIDs: nonsteroidal anti-inflammatory drugs; HRT: hormone replacement therapy.

### Red meat

#### Highest vs lowest intake

**Figure 2:**
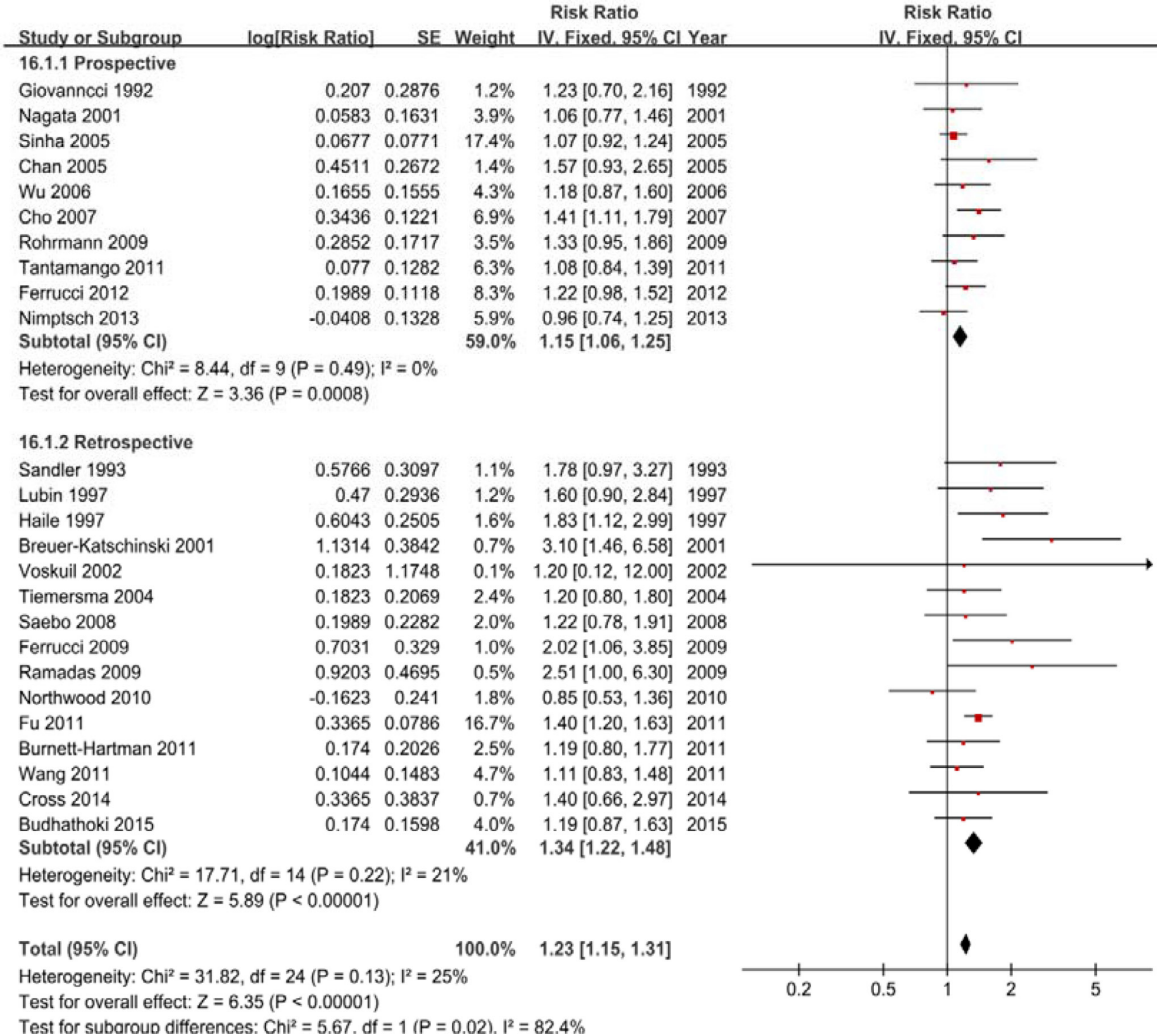
A forest plot of red meat intake and colorectal adenoma incidence

Twenty-five studies were included, and a fixed-effects model yielded positive results (RR = 1.23, 95% CI = 1.15–1.31) with low heterogeneity (*P* = 0.10, *I*^2^ = 28%) (Figure 2, Table 2). Similarly, the subgroup analyses showed that the differences in the RRs were not significant (*P* > 0.05) for sample size, publication year and all adjustments (smoking, alcohol, BMI, physical activity, energy intake, dietary fiber intake, family history of CRC/polyps and nonsteroidal anti-inflammatory drugs) (Supplementary Table 1).

**Table 2:** Analyses of colorectal adenoma locations and subtype analyses of meat for meat intake and colorectal adenoma incidence

	N	RR (95% CI)	*P*_O_	*P*_h_	*I_h_^2^* (%)
**Red meat**					
Total adenoma	25	**1.23 (1.15–1.31)**	**< .01**	.13	25
Proximal colon adenoma	3	1.17 (0.89–1.54)	.27	.51	0
Distal colon adenoma	10	**1.20 (1.09–1.33)**	**< .01**	.39	6
Rectal adenoma	4	1.16 (0.93–1.46)	.19	.76	0
**Red meat/white meat**	**4**	**1.55 (1.10–2.20)**	**.01**	**.03**	**66**
**Processed meat**					
Total adenoma	10	**1.15 (1.07–1.24)**	**< .01**	.10	39
Proximal colon adenoma	0	-	-	-	-
Distal colon adenoma	4	**1.34 (1.11–1.63)**	**< .01**	.31	4
Rectal adenoma	2	0.93 (0.73–1.20)	.58	.39	0
**Subtype analyses of meat**					
Beef	7	**1.45 (1.12–1.89)**	**< .01**	.05	52
Bacon	3	**1.06 (1.03–1.31)**	**.02**	.73	0

NOTE. Boldface indicates statistical significance.

CRA: colorectal adenoma. N: number of included studies. *P*_O_: test for over effect. *P*_h_: *P* value for heterogeneity within each subgroup. *I*_s_^2^: *I*^2^ value for heterogeneity within each subgroup.

#### CRA locations

We further examined the associations between red meat intake and the CRA location. Ten studies were included and the analyses suggested significantly different results, with positive results for distal colon adenoma (RR = 1.21, 95% CI = 1.09–1.34) and negative results for proximal colon adenoma (RR = 1.17, 95% CI = 0.89–1.54) and rectal adenoma (RR = 1.16, 95% CI = 0.93–1.46) (Table 2).

#### Dose-response analysis

Eighteen studies were included, and the results of 1.14 (1.07–1.20) suggested that the CRA incidence increases by 14% for each 100 g/day increase in red meat intake (*P* < 0.01). Furthermore, we checked for nonlinearity of the dose-response relationship and the evidence showed that the best-fitting model was nonlinear model (*P*_nonlinearity_ < 0.01) (Supplementary Figure 3A).

#### Publication bias

The funnel plot (Supplementary Figure 4A) and Egger's test (*P* = 0.94) did not suggest significant evidence of publication bias. The sensitivity analyses of the highest vs lowest categories showed that the changes in the recalculated RRs were not significant, with a range from 1.19 (1.11–1.28) when excluding Fu 2011 [31] (17.1%) to 1.26 (1.18–1.36) when excluding Sinha 2005 [21] (17.8%).

#### Subtype analysis

Beef intake was examined in 7 studies, and the RR of CRA was 1.45 (1.12–1.89) with heterogeneity (*P* = 0.05, *I*^2^ = 52%) (Table 2). Sensitivity analyses of the highest vs lowest categories also showed that the changes in the recalculated RRs were not significant, with a range from 1.31 (1.06–1.63) when excluding Breuer-Katschinski 2001 [16] (8.9%) to 1.59 (1.19–2.12) when excluding Tiemersma 2004 [19] (19.1%).

#### Recurrence

Four studies were included in the comparison of the highest vs lowest categories further stratified analysis for each CRA type. A fixed-effects model yielded null results, with 0.99 (0.84–1.16) for total CRA without heterogeneity (*P* = 0.92, *I*^2^ = 0%), 0.99 (0.82–1.20) for advanced CRA without heterogeneity (*P* = 0.60, *I*^2^ = 0%) and 0.93 (0.75–1.14) for multiple CRA with low heterogeneity (*P* = 0.50, *I*^2^ = 0%) (Supplementary Figure 1, Table 3).

**Table 3:** Analyses of red and processed meat intake and colorectal adenoma recurrence

	N	RR (95% CI)	*P*_O_	*P*_h_	*I_h_^2^* (%)
**Red meat**					
Total adenoma	3	0.99 (0.84–1.16)	.89	.92	0
Advanced adenoma	4	0.99 (0.82–1.20)	.94	.60	0
Multiple adenoma	3	0.93 (0.75–1.14)	.48	.50	0
**Processed meat**					
Total adenoma	3	1.10 (0.94–1.30)	.23	.33	9
Advanced adenoma	4	1.14 (0.95–1.37)	.15	.19	36
Multiple adenoma	3	1.09 (0.73–1.62)	.69	.04	69

N: number of included studies. *P*_O_: test for over effect. *P*_h_: *P* value for heterogeneity within each subgroup. *I*_s_^2^: *I*^2^ value for heterogeneity within each subgroup.

#### Red meat/white meat

Four studies were included in the ratio of red meat to white meat, and a random-effects model yielded significant results (RR = 1.55, 95% CI = 1.10–2.20) with heterogeneity (*P* = 0.03, *I*^2^ = 66%) (Table 2).

### Processed meat

#### Highest vs lowest intake

**Figure 3:**
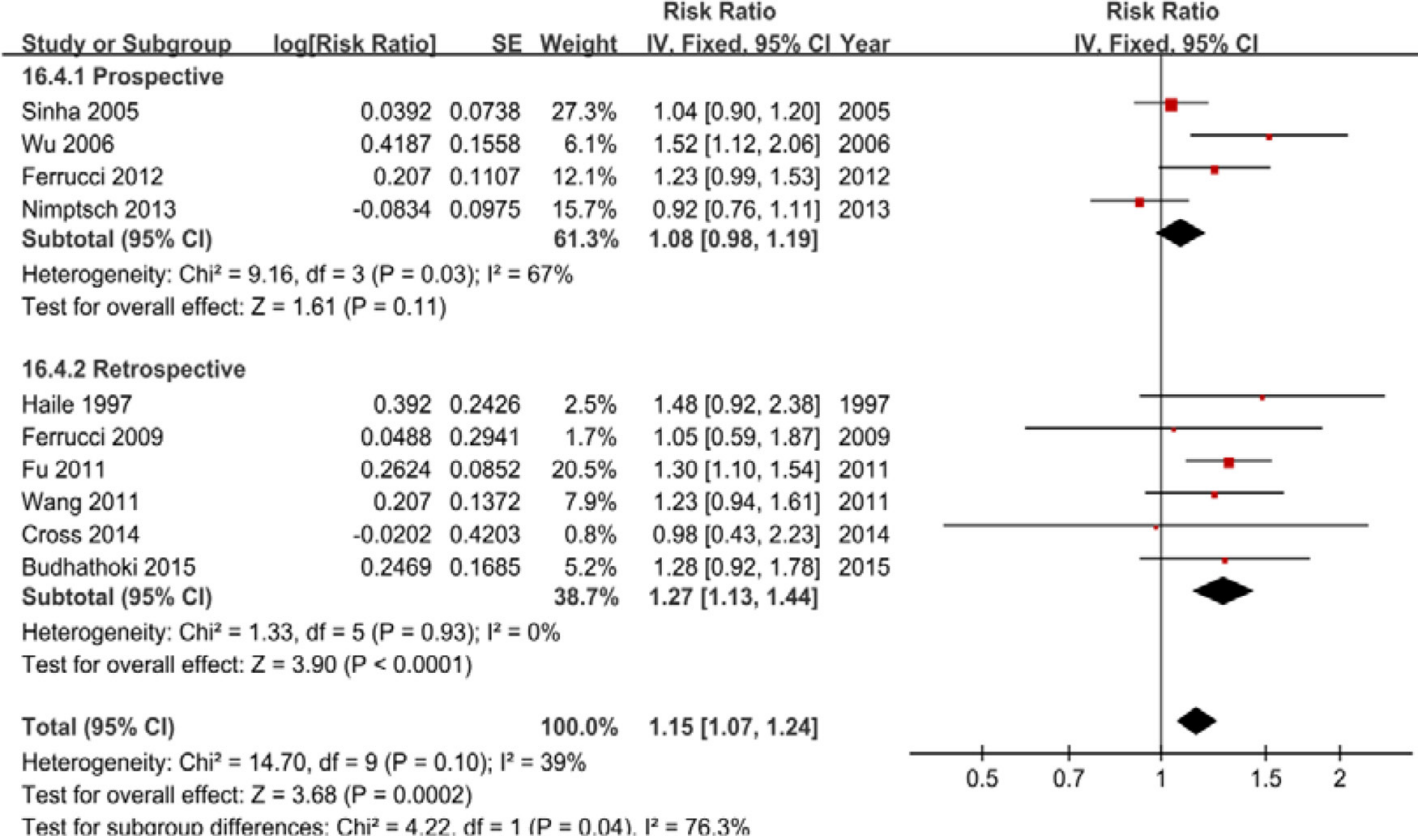
A forest plot of processed meat intake and colorectal adenoma incidence

Ten studies were included, and a fixed-effects model yielded significant results (RR = 1.15, 95% CI = 1.07–1.24) with low heterogeneity (*P* = 0.10, *I*^2^ = 39%) (Figure 3, Table 2). Similarly, the subgroup analyses showed that the differences in the RRs were not significant (*P* > 0.05) for sample size, publication year and all adjustments (smoking, alcohol, BMI, physical activity, energy intake, and nonsteroidal anti-inflammatory drugs) excluded dietary fiber intake and family history of CRC/polyps (Supplementary Table 2).

#### CRA locations

We further examined the associations between processed meat intake and the CRA location. Four studies were included and the analyses suggested significantly different results, with positive results for distal colon adenoma (RR = 1.24, 95% CI = 1.03–1.49) and negative results for rectal adenoma (RR = 0.93, 95% CI = 0.73–1.20) (Table 2). No study examined the association with proximal colon adenoma.

#### Dose-response analysis

Nine studies were included, and the results of 1.27 (1.03–1.50) suggested that the CRA incidence increases by 27% for each 100 g/day increase in processed meat intake (*P* = 0.03). Furthermore, we checked for nonlinearity of the dose-response relationship and the evidence showed that the best-fitting model was nonlinear model (*P*_nonlinearity_< 0.01) (Supplementary Figure 3B).

#### Publication bias

The funnel plot (Supplementary Figure 4B) and Egger's test (*P* = 0.77) did not suggest significant evidence of publication bias. Notably, the sensitivity analyses of the highest vs lowest categories showed that the changes in the recalculated RRs were significant, with a range from 1.12 (1.03–1.22) when excluding Fu 2011 [31] (20.5%) to 1.20 (1.11–1.31) when excluding Nimptsch 2013 [11] (15.7%).

#### Subtype analysis

Bacon intake was examined in 3 studies, and the RR of CRA was 1.16 (1.03–1.31) without heterogeneity (*P* = 0.73, *I*^2^ = 0%) (Table 2). Sensitivity analyses of the highest vs lowest categories also showed that the changes in the recalculated RRs were significant, with a range from 1.32 (0.94–1.84) when excluding Sinha 2005 [21] (86.9%) to 1.16 (1.02–1.31) when excluding Chiu 2004 [36] (2.1%).

#### Recurrence

Four studies were included in the comparison of the highest vs lowest categories when further stratified by CRA type. The results were 1.10 (0.94–1.30) for total CRA with low heterogeneity (*P* = 0.33, *I*^2^ = 9%), 1.14 (0.95–1.37) for advanced CRA with low heterogeneity (*P* = 0.19, *I*^2^ = 36%) and 1.09 (0.73–1.62) for multiple CRA with significant heterogeneity (*P* = 0.04, *I*^2^ = 69%) (Supplementary Figure 2, Table 3).

## DISCUSSION

On the one hand, our findings supported the hypothesis that high intakes of red meat and processed meat increased the CRA incidence. Similarly, the dose-response analyses found positive associations for red meat and processed meat. Furthermore, the results of subgroup analyses that were based on the main adjustment for confounders were consistent for each confounder and similar to the original analyses. Additionally, subtype of analyses for red meat (beef) and processed meat (bacon) yielded the consistent results with the original estimates. We also performed the analyses of CRA locations, which further showed that positive associations were observed in distal CRA for red meat and in proximal CRA for processed meat. We specifically analyzed the ratio of red meat/white meat, and the positive results indicated that the types of meat and the ratio may be associated with CRA risk.

On the other hand, we also examined the associations between red and processed meat intake and CRA recurrence; the analyses indicated that red meat and processed meat intake was not associated with the recurrence of total CRA, advanced CRA and multiple CRA. Overall, our findings highlight the associations between red and processed meat intake and CRA risk, which may be a reference to update the dietary recommendations.

Several potential mechanisms may contribute to the effects. First, the positive associations between red and processed meat intake and CRA risk may be biologically plausible. Cooking red and processed meat is considered one of the major sources of carcinogens, such as heterocyclic amines (HCAs), polycyclic aromatic hydrocarbons (PAHs), nitrate and N-nitroso compounds (NOCs), which are believed to play important roles in the etiology of cancer [37–39] and adenoma [35, 40]. Second, a high iron intake from red meat may play a role in cancer [41] and CRA [42] by promoting the endogenous formation of carcinogenic N-nitroso compounds, causing oxidative damage and lipid peroxidation [43]. Third, positive associations have also been reported to be due to genetically controlled differences. Some specific genetic polymorphisms are considered to be involved in the pathogenesis of CRA [44]. Finally, gut microbial metabolites may be associated with meat intake [45], and bacteriological evidence has revealed possible mechanisms that explain the positive associations to a certain extent [46, 47].

### Study strengths and limitations

There are several limitations in this meta-analysis. First, information on several of the major confounders, such as the intake of vegetable and fruit, could not be provided in all studies. Thus, the findings should be considered carefully due to possible confounding. Second, the different exposure ranges from the lowest to highest categories among included studies contributed to possible heterogeneity. Nevertheless, we adopted the RRs for the comparison of the highest to lowest categories. Additionally, dose-response analyses were conducted to verify the estimate. Third, the cooking methods, storage conditions, production methods and nutrient contents of meat may differ among studies, and the measurement errors to assess meat intake may lead to bias. We cannot thoroughly exclude the potential residual confounding. Finally, the language of studies was limited to English, and several studies with null estimates might not have been reported. Thus, we detected publication bias using the funnel plot, Egger's test, and the sensitivity analysis, which suggested the negligible publication bias.

Our analysis has several strengths. First, this study provided sufficient robust, reliable and current evidence and increased the statistical power based on a substantial sample size and a quantitative synthesis of the eligible data. These data Second, we examined the association between red and processed meat intake and CRA incidence (proximal colon/distal colon/rectum) and recurrence (total/advanced/multiple). We performed subtype analyses of white meat (poultry and fish) to further explore the association. In addition, we conducted subgroup analyses for CRA according to the main risk factors (smoking, alcohol, BMI, energy intake, physical activity, dietary fiber, family history of polyps/CRC and nonsteroidal anti-inflammatory drugs) and the main confounding factors between studies (study design, publication year, sample size and geographic area) to explore the stability of pooled estimates. Third, dose-response analyses were performed to further assess the association rather than simply conducting categorical comparisons. All the independent analyses provided detailed data and increased the statistical power and the strength of our conclusion. Fourth, the study selection and data extraction were performed independently and in duplicate by two authors, which increased the validity of our findings. Finally, the heterogeneity and publication bias of this meta-analysis was negligible, which increased the reliability of our results.

## MATERIALS AND METHODS

### Search strategy

We systematically searched PubMed, EMBASE and Web of Science for studies up to December 2016 using the following search terms: “meats, meat, beef, pork, mutton, veal, lamb, horse, bacon, ham, salami, sausage, hot dogs, lifestyle, food, foods, diet and dietary” in combination with “neoplasm, neoplasms, neoplasia, adenoma, adenomas, cancer, cancers, adenocarcinoma, polyp and polyps”. The two sets were combined individually, and two authors (ZZ and ZY) independently judged the eligibility criteria. Additionally, the reference lists of studies were searched manually to identify eligible literature.

### Study selection

Selection criteria were as follows: studies that diagnosed patients with endoscopy by histological features and biopsy that were consistent with the diagnostic gold standard were included; data that could not be combined were excluded; data that were incomplete were excluded; studies published as original articles were included; pooled analyses, systematic reviews, meta-analyses, narrative reviews, editorials, case reports, letters and comments were excluded; colorectal adenocarcinoma, precancerous lesions and other colorectal tumors were excluded; the included studies were limited to those involving humans and the language was limited to English.

### Study quality and data extraction

Two authors (ZZ and ZY) assessed the quality of included studies independently, and discrepancies in interpretation were resolved by a consensus decision made by the third author (QZ). Study quality was assessed using the Newcastle-Ottawa Scale (NOS) for observational studies [48] and the Cochrane risk of bias tool for randomized controlled trials (RCTs) [49]. A sheet of data extraction was generated for included studies that included the first author, country, publication year, study design, cases, study period, study population, dietary exposure type, dietary assessment method, dietary exposure categories, RRs (95% CI) (highest to lowest), adjusted variables of each study and NOS score.

### Statistical analysis

The STATA version 12.1 (STATA Corporation, College Station, TX) and RevMan5.3 (The Cochrane Collaboration, Oxford, UK) were used for data synthesis and analysis.

A random-effects model was used if there was heterogeneity among studies, and a fixed-effects model was used without heterogeneity. The median or mean level for each category was assigned to each corresponding RR. The non-linear dose-response analysis was conducted using the method described by Greenland et al [50]. The studies that reported RRs with the corresponding 95% CIs for at least 3 quantitative exposure categories were included.

The *I*^2^ statistic (*I*^2^ < 50% was considered low heterogeneity, and *I*^2^ > 50% was considered to indicate substantial heterogeneity) [51] and the Q statistic (*P* < 0.1 was considered representative of significant heterogeneity) were used to detect the heterogeneity among studies. Subgroup analyses were conducted to explore the sources of heterogeneity by study design, publication year, geographic area, sample size and adjustments (smoking, alcohol, BMI, energy intake, physical activity, fiber intake, family history of polyps/CRC and non-steroidal anti-inflammatory drugs).

Publication bias was evaluated using the funnel plot, Egger's test [52] and a sensitivity analysis. *P* < 0.1 of Egger's test was considered significant publication bias. The sensitivity analysis was performed to investigate the influence of each study on the pooled risk estimate by removing one study in turn.

## CONCLUSIONS

The present analysis provided evidence that the intake of red meat and processed meat was associated with an increased incidence of CRA. No associations were found between red meat and processed meat intakes and CRA recurrence.

## SUPPLEMENTARY MATERIALS FIGURES AND TABLES


