# Supplementary material for: Association between red and processed meat intake and colorectal adenoma incidence and recurrence: a systematic review and meta-analysis
Source: Oncotarget. 2017 Dec 21;9(64):32373–82. doi: 10.18632/oncotarget.23561 (PMC6122348; doi:10.18632/oncotarget.23561)
Supplement: Supplementary file 1 [file oncotarget-09-32373-s001.pdf]

Association between red and processed meat intake and colorectal adenoma incidence and recurrence: a systematic review and meta-analysis

SUPPLEMENTARY MATERIALS

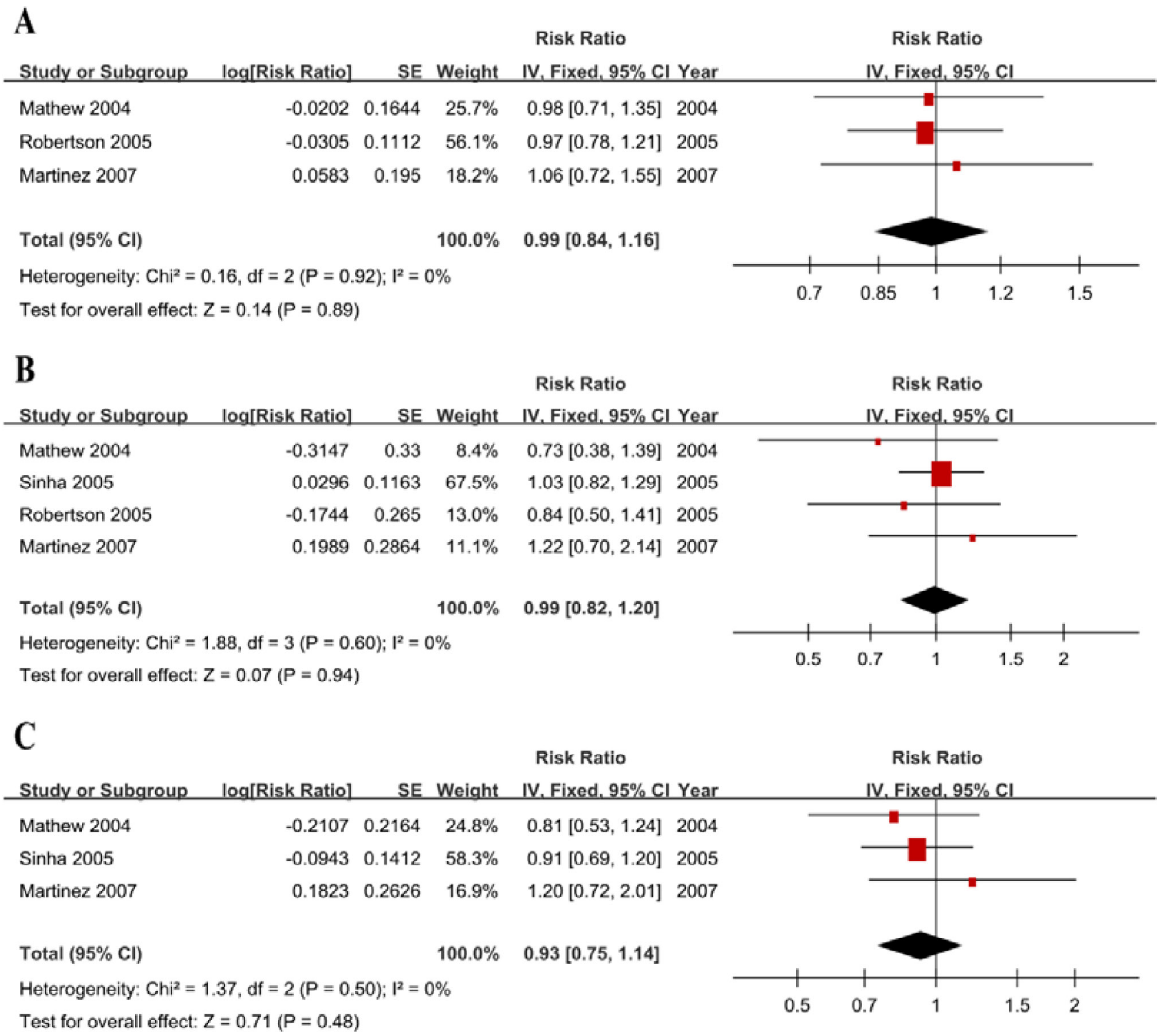

Supplementary Figure 1: Forest plots of red meat intake and colorectal adenoma recurrence. (**A**) Total colorectal adenoma. (**B**) Advanced colorectal adenoma. (**C**) Multiple colorectal adenoma.

A

Study or Subgroup	log[Risk Ratio]	SE	Weight	Risk Ratio IV, Fixed, 95% CI	Year
Mathew 2004	-0.0834	0.155	27.9%	0.92 [0.68, 1.25]	2004
Robertson 2005	0.1398	0.112	53.4%	1.15 [0.92, 1.43]	2005
Martinez 2007	0.2564	0.1894	18.7%	1.29 [0.89, 1.87]	2007
**Total (95% CI)**			**100.0%**	**1.10 [0.94, 1.30]**	

Heterogeneity: Chi² = 2.21, df = 2 (P = 0.33); I² = 9%

Test for overall effect: Z = 1.21 (P = 0.23)

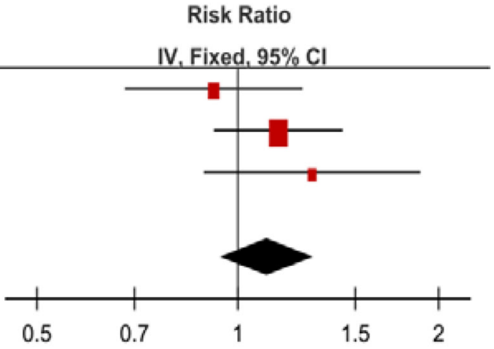

B

Study or Subgroup	log[Risk Ratio]	SE	Weight	Risk Ratio IV, Fixed, 95% CI	Year
Mathew 2004	-0.1985	0.3294	8.1%	0.82 [0.43, 1.56]	2004
Robertson 2005	0.5596	0.274	11.6%	1.75 [1.02, 2.99]	2005
Sinha 2005	0.0583	0.1126	68.9%	1.06 [0.85, 1.32]	2005
Martinez 2007	0.3853	0.2775	11.4%	1.47 [0.85, 2.53]	2007
**Total (95% CI)**			**100.0%**	**1.14 [0.95, 1.37]**	

Heterogeneity: Chi² = 4.70, df = 3 (P = 0.19); I² = 36%

Test for overall effect: Z = 1.42 (P = 0.15)

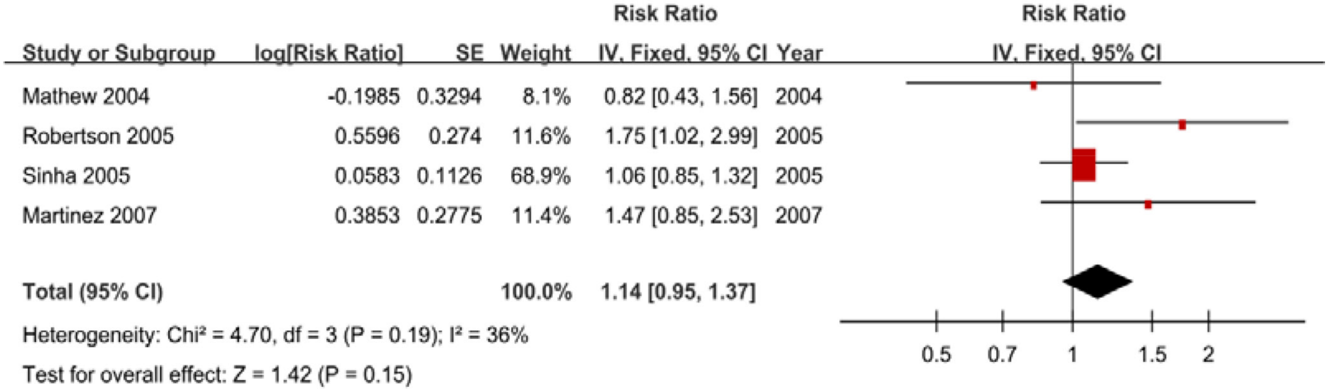

C

Study or Subgroup	log[Risk Ratio]	SE	Weight	Risk Ratio IV, Random, 95% CI	Year
Mathew 2004	-0.1278	0.2127	32.1%	0.88 [0.58, 1.34]	2004
Sinha 2005	-0.1054	0.1356	40.4%	0.90 [0.69, 1.17]	2005
Martinez 2007	0.6043	0.2597	27.5%	1.83 [1.10, 3.04]	2007
**Total (95% CI)**			**100.0%**	**1.09 [0.73, 1.62]**	

Heterogeneity: Tau² = 0.09; Chi² = 6.38, df = 2 (P = 0.04); I² = 69%

Test for overall effect: Z = 0.40 (P = 0.69)

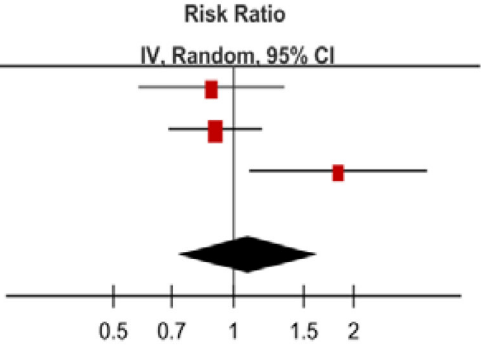

Supplementary Figure 2: Forest plots of processed meat intake and colorectal adenoma recurrence. (**A**) Total colorectal adenoma. (**B**) Advanced colorectal adenoma. (**C**) Multiple colorectal adenoma.

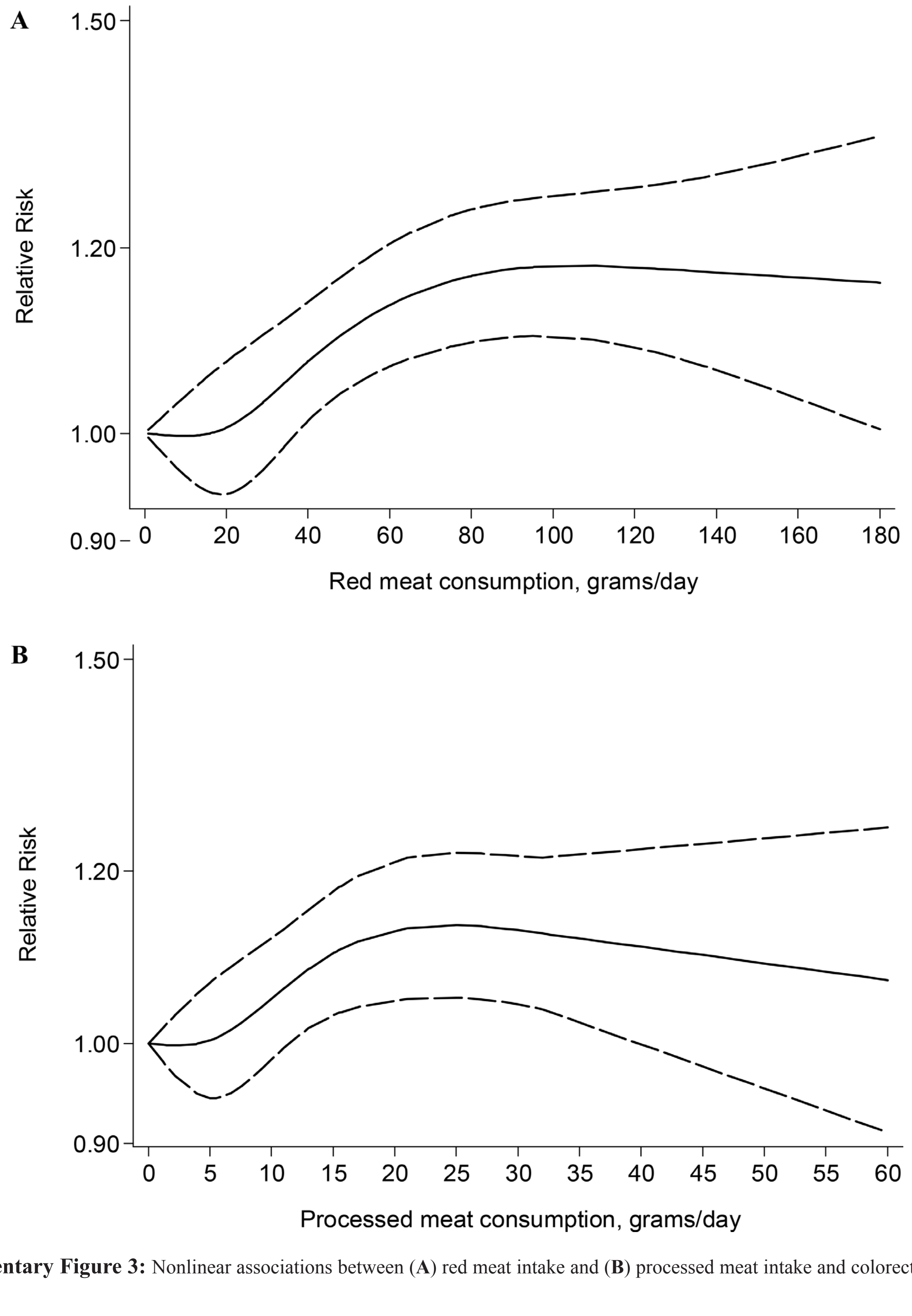

Supplementary Figure 3: Nonlinear associations between (**A**) red meat intake and (**B**) processed meat intake and colorectal adenoma incidence.

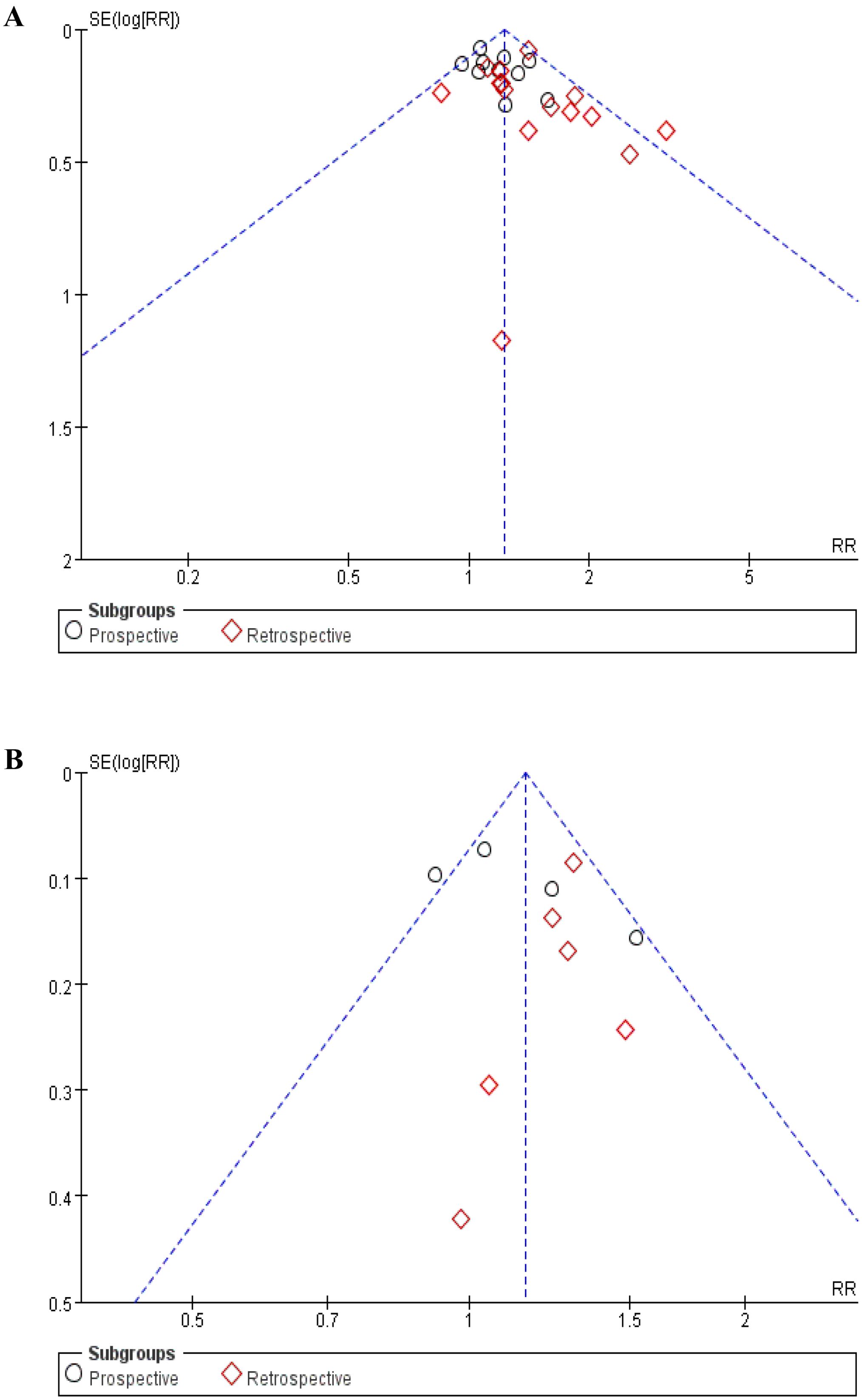

Supplementary Figure 4: Funnel plots evaluating publication bias of red and processed meat intake and colorectal adenoma incidence. (**A**) Red meat. (**B**) Processed meat. SE: standard error. RR: relative risk.

Supplementary Table 1: Subgroup analyses of red meat intake for Highest vs Lowest comparisons and CRA incidence

Subgroups	Red meat				
	N	RR (95% CI)	P_O	P_h	I_h^2 (%)
**All studies**	25	**1.23 (1.15–1.31)**	**< .01**	.13	25
**Geographic area**					
**America**	15	**1.22 (1.14–1.31)**	**< .01**	.15	28
**Europe**	6	**1.25 (1.03–1.52)**	**.02**	.14	40
**Asia**	4	1.22 (1.00–1.50)	.05	.26	25
**Sample size**					
**≥ 500**	11	**1.21 (1.13–1.30)**	**< .01**	.27	18
**< 500**	14	1.27 (1.12–1.44)	< .01	.12	32
**Publication year**					
**2005 or later**	17	**1.21 (1.13–1.29)**	**< .01**	.21	21
**Before 2005**	8	1.38 (1.15–1.65)	< .01	.20	29
**Adjustments Smoking**					
**Yes**	16	**1.22 (1.14–1.30)**	**< .01**	.13	30
**No**	9	1.28 (1.08–1.53)	< .01	.25	21
**Alcohol**					
**Yes**	15	**1.21 (1.13–1.30)**	**< .01**	.13	30
**No**	10	1.29 (1.10–1.51)	< .01	.26	20
**BMI**					
**Yes**	16	**1.29 (1.20–1.40)**	**< .01**	.09	34
**No**	9	1.11 (0.99–1.23)	.06	.88	0
**Energy intake**					
**Yes**	17	**1.25 (1.16–1.34)**	**< .01**	.04	41
**No**	8	1.16 (1.01–1.33)	.04	.82	0
**Physical activity**					
**Yes**	16	**1.23 (1.15–1.32)**	**< .01**	.12	30
**No**	9	1.20 (1.03–1.41)	.02	.24	23
**Dietary fiber**					
**Yes**	8	**1.21 (1.10–1.32)**	**< .01**	.16	33
**No**	17	1.24 (1.14–1.35)	< .01	.17	24
**Family history of CRC/polyps**					
**Yes**	10	**1.21 (1.09–1.33)**	**< .01**	.50	0
**No**	15	1.24 (1.14–1.35)	< .01	.06	40
**NSAIDs**					
**Yes**	12	**1.24 (1.15–1.33)**	**< .01**	.12	34
**No**	13	1.19 (1.05–1.35)	< .01	.25	19

NOTE. Boldface indicates statistical significance.
N: number of included studies. CRA: colorectal adenoma. CRC: colorectal cancer. BMI: body mass index. NSAIDs: non-steroidal anti-inflammatory drugs. P_O: test for over effect. P_h: P value for heterogeneity within each subgroup. I_s^2: I^2 value for heterogeneity within each subgroup.

Supplementary Table 2: Subgroup analyses of processed meat intake for Highest vs Lowest comparisons and CRA incidence

Subgroups	Processed meat				
	N	RR (95% CI)	P_O	P_h	I_h^2 (%)
**All studies**	10	**1.15 (1.07–1.24)**	**< .01**	.10	39
**Geographic area**					
**America**	9	**1.15 (1.06–1.24)**	**< .01**	.07	44
**Europe**	0	-	-	-	-
**Asia**	1	1.28 (0.92–1.78)	.14	-	-
**Sample size**					
**≥ 500**	7	**1.18 (1.04–1.33)**	**.01**	.04	55
**< 500**	3	1.23 (0.88–1.72)	.22	.56	0
**Publication year**					
**2005 or later**	9	**1.16 (1.04–1.30)**	**< .01**	.09	41
**Before 2005**	1	1.48 (0.92–2.38)	.11	-	-
**Adjustments Smoking**					
**Yes**	10	**1.18 (1.06–1.31)**	**< .01**	.54	0
**No**	0	-	-	-	-
**Alcohol**					
**Yes**	7	**1.13 (1.04–1.22)**	**< .01**	.13	40
**No**	3	1.45 (1.14–1.86)	.20	.90	0
**BMI**					
**Yes**	8	**1.18 (1.05–1.32)**	**< .01**	.22	26
**No**	2	1.23 (0.85–1.77)	.28	.03	79
**Energy intake**					
**Yes**	8	**1.17 (1.04–1.33)**	**< .01**	.05	51
**No**	2	1.23 (0.91–1.68)	.55	-	-
**Physical activity**					
**Yes**	10	**1.18 (1.06–1.31)**	**< .01**	.54	0
**No**	0	-	-	-	-
**Dietary fiber**					
**Yes**	5	1.11 (0.99–1.24)	.07	.53	0
**No**	5	1.21 (1.02–1.45)	.03	.03	63
**Family history of CRC/polyps**					
**Yes**	6	**1.16 (0.97–1.39)**	**.10**	.09	47
**No**	4	1.19 (1.03–1.38)	.02	.16	42
**NSAIDs**					
**Yes**	8	**1.18 (1.04–1.34)**	**.01**	.05	51
**No**	2	1.20 (0.93–1.55)	.16	.61	0

NOTE. Boldface indicates statistical significance.

N: number of included studies. CRA: colorectal adenoma. CRC: colorectal cancer. BMI: body mass index. NSAIDs: nonsteroidal anti-inflammatory drugs. P_O: test for over effect. P_h: P value for heterogeneity within each subgroup. I_s^2: I^2 value for heterogeneity within each subgroup.
